# Profile: Vadu Health and Demographic Surveillance System Pune, India

**DOI:** 10.7189/jogh.09.010202

**Published:** 2019-06

**Authors:** Rutuja Patil, Sudipto Roy, Vijendra Ingole, Tathagata Bhattacharjee, Bharat Chaudhary, Pallavi Lele, Siddhivinayak Hirve, Sanjay Juvekar, on behalf of VaduHDSS team

**Affiliations:** 1 KEM Hospital Research Centre Pune (KEMHRC), Vadu Rural Health Program, India; 2 Usher Institute, College of Medicine and Veterinary Medicine, University of Edinburgh, Edinburgh UK; 3 INDEPTH Network, Accra, Ghana; 4 ISGlobal, Barcelona, Spain

Vadu Health and Demographic Surveillance System (VaduHDSS), a surveillance system initiated in 2002, is run by Vadu Rural Health Program of the KEMHRC Pune. Every six month, the VaduHDSS collects data on pregnancies, births, marriages, migrations, deaths and assesses causes of deaths using verbal autopsies in a population of 160000 individuals residing in about 48000 households in 22 villages. By virtue of its longitudinal data generation practices the VaduHDSS facilitates national and globally relevant health research to generate evidence for informed health policy decisions.

Availability of reliable empirical health and demographic data are crucial to understand the burden and trends of health conditions/diseases and plan health interventions. Typically, such data are obtained from sample populations, which is then used to calculate estimates for the larger community with the help of statistical analyses and modeling techniques. Though these methods are robust and can result in fairly accurate estimates, these are heavily dependent on sampling techniques and sample characteristics and can have significant errors in estimates. One method to improve this is to establish a health and demographic surveillance system (HDSS). An HDSS involves an ongoing, long-term monitoring of vital events, demographic characteristics and health status of a geographically defined population. The complete population within an HDSS is under surveillance and thus relevant data are available for all its individuals. An HDSS enables us to monitor trends in diseases and conditions and track mortality rates and help to fill gaps in health-related data. Additionally, it provides a platform to conduct health research studies.

Here we present the profile of an HDSS in a rural part of Pune district, India, set up by KEM Hospital Research Centre Pune. We describe the surveillance system and report some key findings from it, including the advantages of a HDSS.

## WHY WAS THE VADUHDSS SET UP?

KEM Hospital (KEMH), a 106-year-old multi-specialty, 550 bedded, tertiary care hospital in Pune city [1], started providing primary health care to rural communities in and around Vadu village in the early part of the decade of 1970 as the area lacked adequate health care facilities. It started a secondary level health facility with support from public health systems by establishing a 30-bedded hospital named Shirdi Sai Baba Rural Hospital in 1987. In 1988, KEMH added a community-based outreach program [2], termed as Vadu Rural Health Program (VRHP), catering to the primary health care needs of the surrounding rural population simultaneously. To enhance the quality of health care, KEM Hospital Research Centre (KEMHRC), a sister concern of KEMH which focuses on research, started conducting health research in VRHP area in 1983 [3]. With an increasing realization of the importance of social determinants of health, there was a felt-need to better understand the complex interaction between socio-demographic characteristics and health at the individual and population level. With this in view, the Vadu Health and Demographic Surveillance System (VaduHDSS) was initiated in a geographically contiguous population of 50000 individuals covering 22 villages in 2002, with seed grant from the Gates Institute for Population and Reproductive Health, Johns Hopkins University Bloomberg School of Public Health. The overarching objectives of VaduHDSS are:

1. To continuously monitor the demographic dynamics in a rural population in terms of births, deaths, cause of death (COD) and migration
2. To facilitate clinical, epidemiological, socio-behavioural and intervention health research
3. To establish a platform for evaluation of public health interventions
4. To generate evidence for informed health policy decisions

The HDSS facilitates national and globally relevant health research in line with its mission to *“Provide evidence-based, sustainable and rational health care solutions for the rural population using globally relevant community-based ethical research”*.

## WHAT DOES IT COVER NOW?

**Figure 1:**
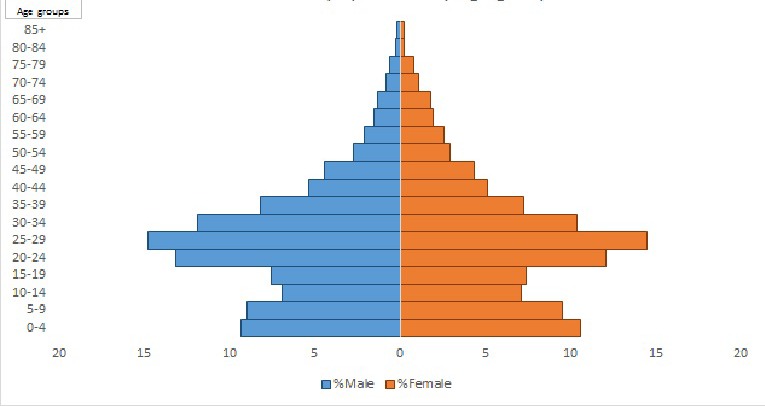
Population pyramid for Vadu Health and Demographic Surveillance System (VaduHDSS) area.

**Figure 2:**
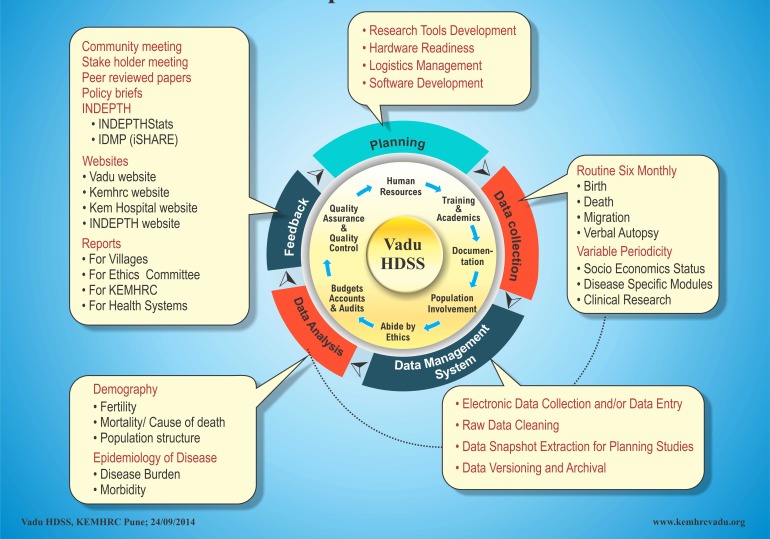
Vadu Health and Demographic Surveillance System (VaduHDSS) conceptual framework.

VaduHDSS currently includes 160 000 individuals residing in 22 villages of Shirur and Haveli Block of Pune district in Maharashtra, India. VaduHDSS has experienced a rise in the population from an initial 50000 in 2002 to 160000 in 2018. While VaduHDSS area was primarily an agrarian community in 2002, today a significant proportion of population is employed in industries. Rapid growth of industries has resulted in newer occupational opportunities [4,5] leading to in-migration of younger generation from all over the country, which is indicated by the bulging mid-section of the population pyramid (**Figure 1**). The following conceptual framework (**Figure 2**) describes how VaduHDSS is inclusive in its approach and how the population is central to its activities.

**Figure 3:**
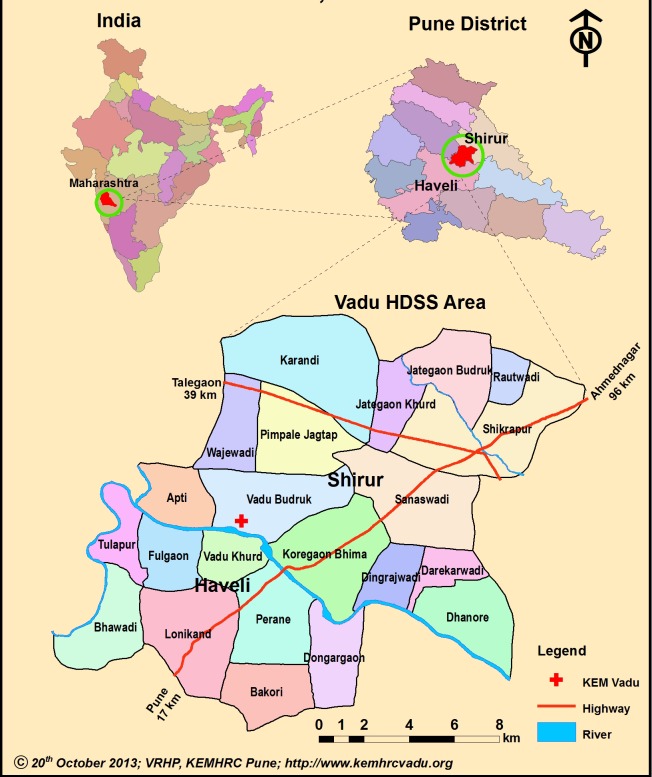
Vadu Health and Demographic Surveillance System (VaduHDSS) area map.

VaduHDSS (latitude 18°30 to 18°47N & longitude 73°58 to 74°12E) covers a geographical area of 232km^2^ with an average altitude of 560m (**Figure 3**). There are three seasons: Monsoons June-October with mean annual rainfall of 720mm, Winter October-February with minimum temperatures between 7-9°C and Summer February-May with maximum temperatures of 40-42°C.

We collect data from local residents who are either native or those who migrate to this area with an intention to stay for more than three months. All households in VaduHDSS area are visited once every six months during which data are collected on births, deaths, marriage, migrations, pregnancy status and outcomes of every woman in reproductive age group and self-reported common illnesses. Verbal Autopsy (VA) is conducted for every death.

**Figure 4:**
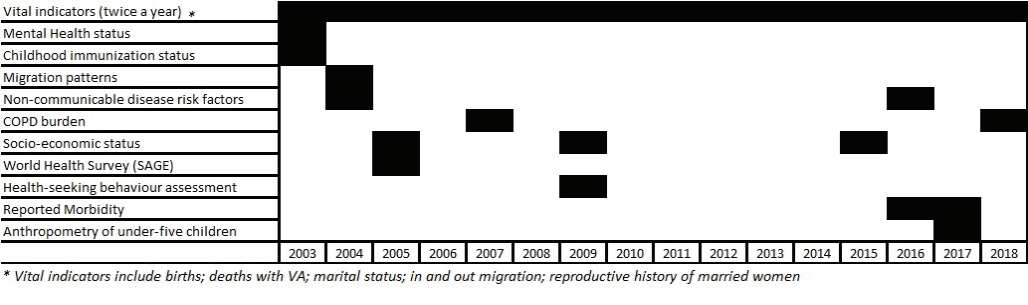
Timelines describing cohorts development at Vadu Health and Demographic Surveillance System (VaduHDSS).

We have established some sub-cohorts within VaduHDSS to look at specific health conditions. A cohort of 15000 children aged <5 years to assess nutritional status using anthropometry was established in 2017. We plan to follow each child till the individual reaches 12 years. A Non-Communicable Diseases Risk Factor surveillance (NCD-RF cohort of 2400 individuals) was conducted in 2004 with support from WHO-NCD division and the same cohort was followed up, with institutional support, in 2017 to determine changes in NCD-RF [6,7]. Under the study of Global Ageing (WHO-SAGE) a cohort of 440 elderly individuals of 55 years and older was established to know cognitive elderly functioning and health status assessment [8,9]. Another cohort to understand Burden of Obstructive Lung Disease (BOLD) was established in 2007 to estimate COPD burden/ its phenotypes using spirometry & clinical assessment. This cohort of 3400 adults over 25 years of age, was established in collaboration with Imperial College London [10,11]. This cohort is now being followed up to determine the progress of COPD disease in these individuals. A cohort of 6000 children under 15 years of age is currently being followed up to estimate the burden of enteric fever [12]. Timelines describing cohorts developed are described in the Gantt chart (**Figure 4**).

## WHAT HAS BEEN MEASURED AND HOW HAVE HDSS DATABASES BEEN CONSTRUCTED?

VaduHDSS is a member of International Network for the Demographic Evaluation of Populations and Their Health (INDEPTH) that conducts longitudinal surveillance for monitoring and evaluation of population health in low and middle-income countries. The routine surveillance round collects demographic information as per **Table 1**. Additionally, all deaths within HDSS area are recorded and subjected to VA using an adapted version of WHO VA tools followed by physician assigned COD using ICD10 classification (International Classification of Diseases-Version10). We are now migrating to electronic assessment of COD. Changes such as new location and household change are registered and updated in the database.

**Table 1:** Demographic information collected during HDSS round

Data forms	Information collected
Individuals	Name, Individual ID, occupation, education, age, marital status, relationship with household head, date of birth
Births	Name, ID, sex of child, date of birth, mother’s and father’s ID, place of birth, birth weight
Deaths	Causes of deaths, date of deaths, place of deaths, VA
Immigration	Date of in-migration, reason for migration
Outmigration	Date of out-migration, reason for migration, destination of migration
Pregnancy	Outcome of pregnancy, date of last cycle

ID – identification, VA – verbal autopsy

Data collection at VaduHDSS is done by its Field Research Assistants (FRA) who are local residents of the VaduHDSS area. The FRAs are trained to collect research data from the community, using ethical practices. This training has in turn enhanced the capacities of community in using technology and understanding health. This data are further checked for quality on field by the field research supervisors and later by the data managers once the data are uploaded on the servers on a weekly basis.

**Figure 5:**
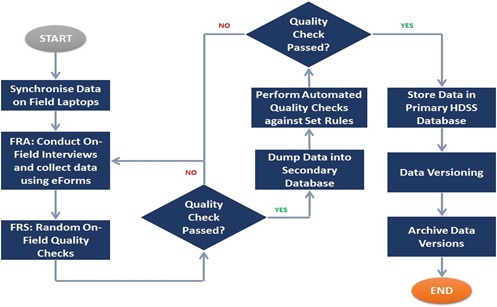
Vadu Health and Demographic Surveillance System (VaduHDSS) data flow.

The first round of VaduHDSS data collection was in 2002. Initial rounds of HDSS data collection were paper-based followed by data entry using a *FoxPro* based software. With increasing availability of technology, we started electronic data capture using laptops in 2007. This was done using an application developed using *PHP My Admin* software for front-end application and *MySQL* database. Shifting from paper based to electronic data capture helped up to reduce data-entry errors and other errors due to built-in quality checks. Moreover, it has substantially reduced time and cost of data collection. In 2013, we started data collection on electronic tablet using an android application based on *SQLite* software, developed in house. Current data flow is described in **Figure 5**. From July 2019 we plan to move to more opensource platform “Survey solutions” for long term sustainability. Since 2009 we have initiated an open data access policy. Our data are available in public domain on the INDEPTH-iShare website along with the metadata, thus providing anonymized longitudinal microdata to researchers across the globe.

## HOW HAS VADUHDSS DATA BEEN USED

VaduHDSS has contributed to national programs on immunization, tubectomy, vasectomy, tuberculosis control, childhood disability identification program and development of community health volunteer schemes. Examples of some study findings which have made their way to implementation by the public health system include Sprinkles micronutrient powder [13,14] and iron fortified wheat flour [15] as well as vaccine studies on Rota virus vaccine [16-21]. Studies conducted in VaduHDSS, in addition to providing evidence to government, have created significant scientific literature as outcome of its studies [22]. A list of publications is available on www.kemhrcvadu.org/index.php/academics/publications.

## KEY FINDINGS FROM VADUHDSS

VaduHDSS has experienced a steep rise in the population since 2002 leading to a current population density of 636 people per square km. Other demographic characters for the years 2009, 2013 and 2017 along with the comparative information available for contemporary time period from the Indian government records (NITI Aayog) are described in details in **Table 2**.

**Table 2:** Demographic characteristics of VaduHDSS in 2009, 2013 and 2017 compared to the government figures for state

		2009		2013		2017	
		**Vadu HDSS**	**NITI Aayog (Maharashtra)**	**Vadu HDSS**	**NITI Aayog (Maharashtra)**	**Vadu HDSS**	**NITI Aayog (Maharashtra)**
2	Mid year population	97996	NA	131105	NA	148256	NA
4	Households - mid year count	30293	NA	46594	NA	48347	NA
5	Sex ratio at birth - per 1000	812	893 (2009-11)	910	902 (2011-13)	884	878 (2013-15)
7	Population density per sq km	422	NA	565	NA	636	NA
8	Crude birth rate	17.66	17.66 (2016)	17.6	16.5 (2013)	20	15.9 (2016)
9	Crude death rate	4	6.7 (2009)	4	6.2 (2013)	3	5.9 (2016)
10	Total fertility rate	1.5	1.9 (2009)	1.6	1.8 (2013)	1.6	1.8 (2016)
11	Neonatal mortality rate per thousand live births	11.62	18 (2011)	3.47	17 (2013)	3.03	NA
12	Infant mortality rate	12	31 (2009)	6	24 (2013)	6	19 (2016)
13	Under five mortality rate	18	28 (2011)	10	26 (2013)	8	18 (2015)
14	Life expectancy at birth (male)	77	67.9 (2006-10)	72	69.4 (2009-13)	77	69.9 (2010-14)
15	Life expectancy at birth (female)	76	71.9 (2006-10)	77	73.4 (2009-13)	79	73.6 (2010-14)

NA – not available

In 2017, about 56% individuals were males which has been about the same since 2009. Around 26% of the total population had completed primary school. Children aged <15 years constituted 25% and those <5 years constituted 10% of the population while only 4% of the population was >65 years. Other background characteristics for three time points, ie, 2009, 13 and 17 are given in **Table 3**.

**Table 3:** Background characteristics in years 2009, 2013 and 2017

Variable	Categories	2009		2013		2017	
		**Frequency**	**Percent**	**Frequency**	**Percent**	**Frequency**	**Percent**
Sex	Female	44 572	43.41	55 839	41.46	65 679	43.72
	Male	58 098	56.59	78 854	58.54	84 558	56.28
	Total	**102 670**	**100.00**	**134 693**	**100.00**	**150** **237**	**100.00**
Education completed	<Primary school			29 364	13.09	45 008	12.84
	Primary school			59 369	26.47	90 256	25.74
	Secondary school			43 613	19.44	63 809	18.2
	High school			33 804	15.07	60 290	17.2
	Graduation			12 918	5.76	28 497	8.13
	Post-graduation			3197	1.43	6326	1.80
	No schooling			41 850	18.66	56 297	16.06
	Others			197	0.09	140	0.04
	Total			**224 312**	**100.00**	**350 623**	**100.00**
Marital status	Currently married			111 087	49.52	174 256	50.45
	Currently re-married			187	0.08	532	0.15
	Separated			525	0.23	900	0.26
	Divorced			91	0.04	158	0.05
	Widowed			7470	3.33	9727	2.82
	Never married			104 535	46.6	158 089	45.77
	Others			417	0.18	1745	0.51
	Total			**224 312**	**100.00**	**345 407**	**100.00**
Age categories female	Less than 1 y	1 008	2.26	1416	2.54	1449	2.21
	1-4.99 y	3 634	8.15	4797	8.59	5711	8.70
	5-14.99 y	7 752	17.39	9215	16.50	10 342	15.75
	15-28.99 y	14 711	33.01	18 130	32.47	20 460	31.15
	29-64.99 y	15 546	34.88	19 982	35.79	24 728	37.65
	More than 65 y	1 921	4.31	2 299	4.12	2989	4.55
	Total	**44 572**	**100**	**55 839**	**100.00**	**65 679**	**100.00**
Age categories male	Less than 1 y	1 243	2.14	1518	1.93	1618	1.91
	1-4.99 y	4 336	7.46	5641	7.15	6426	7.60
	5-14.99 y	9 390	16.16	11 413	14.47	12 693	15.01
	15-28.99 y	21 877	37.66	31 553	40.01	28 740	33.99
	29-64.99 y	19 226	33.09	26 410	33.49	32 215	38.10
	More than 65 y	2 026	3.49	2319	2.94	2866	3.39
	Total	**58 098**	**100.00**	**78 854**	**100.00**	**84 558**	**100.00**
Age categories both sexes	Less than 1 y	2 251	2.19	2934	2.18	3067	2.04
	1-4.99 y	7 970	7.76	10 438	7.75	12 137	8.08
	5-14.99 y	17 142	16.70	20 628	15.31	23 035	15.33
	15-28.99 y	36 588	35.64	49 683	36.89	49 200	32.75
	29-64.99 y	34 772	33.87	46 392	34.44	56 943	37.90
	More than 65 y	3 947	3.84	4618	3.43	5855	3.90
	Total	**102 670**	**100.00**	**134 693**	**100.00**	**150 237**	**100.00**

y – year

Morbidity surveys indicated that 1.7% of total population reported fever in past two weeks, while 1% reported fever with cough. Of the population, 0.6% had diarrhea in previous two weeks. Hypertension, diabetes and asthma were significantly higher in individuals aged >50 years with proportions of 5.55%, 5.14% and 1.86% respectively. These are reported to be significantly highest in farmers, while fever was reported to be highest in individuals working in manufacturing/service industry.

In addition to this, in a nutritional survey in under-five children, mid-upper arm circumference measurements indicated that 16% of children were mildly undernourished, 5% were moderately undernourished and 1.2% were severely undernourished. Analysis of data collected from NCD and SAGE cohorts in 2005-06 almost two-thirds of men and half of women in VaduHDSS chewed tobacco daily [23]. Considerable proportion of the survey sample in VaduHDSS area has high blood pressure both in men (25.5%) and women (21.6%) [24]. Reported prevalence of physical inactivity was higher (58%) [25] and prevalence of chronic conditions was also found to be high in both men (28.6%) and women (35.8%) [6]. Prevalence of self-reported chronic condition in adults over 50 years of age in 2005-06 was 28.6 in men and 35.8 in women [15,16].

Non-communicable diseases are the most common causes of death in VaduHDSS population. This is reflective of the changing epidemiology of common diseases in India, even in rural areas. The second highest COD is accidental deaths owing to increasing availability and use of vehicles in the newly industrialized area and changing socio-economic status. Moreover, mortality is associated with ambient temperature in VaduHDSS and has been analyzed in context of various confounding factors [26,27].

VaduHDSS data indicates that between 2004 and 2016 proportion of agricultural cultivators reduced from 22.58% to 13.69% while agricultural landowners decreased from 61% to 25% indicating reduction in people’s dependency on agriculture and increased inclination towards industrial jobs as indicated by the increased proportion of individuals working in small and medium scale industries from 36% in 2004 to 44% in 2015.

The socio-economic development of this area is reflected in increase in use of flushed toilet facilities by 7% and increase in use of electricity by 11% between 2004-05 and 2014-15. Additionally, proportion of households using Liquefied Petroleum Gas for cooking increased from 55% to 77.45% in ten years. One important change that this area has seen since 2002 is the growth of mixed health care delivery sector, with both private and government health facilities providing health services.

Since 2002 nearly 68 research studies have been conducted based on the VaduHDSS. VaduHDSS has a strong interest in health of women and children. A study conducted in 2012 on marriage preparedness among adolescent girls reported that almost 90% of women marry between the ages of 18 and 21 years. The most common factors associated with early marriage were poor economic conditions, societal pressure and concern about the security of girls due to perceived unsafe environment. The studies at VaduHDSS on water, sanitation and hygiene (WASH) infrastructure and practices in women conducted in 2016 reported that prevalence of open defecation in HDSS area is low and most people have access to WASH installations in or close to their living quarters.

VaduHDSS participation in conducting large community-based trials for meningitis, measles, typhoid, rotavirus and pneumococcal vaccines is one important contribution towards the development of safe, efficacious and affordable vaccines global use [15-20,22-25,28-30]. VaduHDSS has always led the technology portfolio within INDEPTH network and was instrumental in the development of online data repository iSHARE (www.INDEPTH-iSHARE.org). VaduHDSS utilizes technology for providing health services to rural population at remote station through its micro-health centre (MHC). MHC is a ready-to-use integrated health system for delivery of community-based primary health care. MHC generates medical data for a national database using cloud-enabled health care database management system [31].

## IMPORTANT COLLABORATIONS

VaduHDSS, since inception, being part of INDEPTH Network has mutually supported its activities. Other than the regular HDSS, VaduHDSS has lead nearly ten working groups including the Non-communicable diseases, communication and policy, indoor air pollution, and data sharing groups. VaduHDSS has leveraged INDEPTH partnership for developing and strengthening abilities to develop and work on multicentric research projects projects.

VaduHDSS has developed a portfolio on research in respiratory health over the past decade and is currently a part of RESPIRE a global health research unit focusing on respiratory health [32,33], that aims to conduct research to reduce mortality and morbidity due to respiratory conditions. In past, estimating the disease burden of influenza [28,29,34] and determining the incidence of acute respiratory infections [35] were the aims of two studies in the acute respiratory illness domain. Regarding chronic respiratory conditions, VaduHDSS has conducted a study to estimate the prevalence of obstructive lung diseases [10,11,36] in the area under the BOLD collaboration. VaduHDSS has also conducted research into the health effects of indoor air pollution (IAP), including estimating the prevalence of biomass fuel use and an intervention study using improved cook-stoves to reduce IAP [37-41]. VaduHDSS` experience in respiratory health research and a well-established research infrastructure offers a conducive environment to conduct research within RESPIRE. Additionally, the ability of HDSS to support respiratory health research has been observed in the successful conduct of the ANISA study in Bangladesh [42].

## STRENGTHS AND CHALLENGES

VaduHDSS has its strength embedded in its field staff who are local villagers, This, in other words mean that at times, the field staff are members of participating households. The Field staff in VaduHDSS are known as the Field Research Assistants (FRA) who themselves are trained to conduct few aspects of community based research. They are trained with good clinical practices techniques so as to ensure ethical conduct during data collection [22]. The second strength is its data management team that is capable of generating quality data collection tools, timely cleaning and provision of data for researchers to use and timely data sharing in public domain [43,44].

The analysis and use of the voluminous amounts of data generated in the HDSS can be challenging. This is one reason VaduHDSS encourages open data sharing, described in the next section and collaborations throughout the world to use the data.

## DATA SHARING AND COLLABORATION

The data are shared within institution and with outsiders. Within institution, the data are shared for data analysis, publication, as sampling frame for newer studies and for planning studies. The data for outsiders are shared directly in public domain using INDEPTH Data Repository through iSHARE along with the metadata following the Data Documentation Initiative (DDI3) standards (www.INDEPTH-iSHARE.org).
